# Bone density loss after risk reducing salpingo-oophorectomy

**DOI:** 10.1186/1897-4287-10-S2-A79

**Published:** 2012-04-12

**Authors:** L Andrews, B Zielony, C White

**Affiliations:** 1 Hereditary Cancer Clinic, Prince of Wales Hospital, Randwick, Australia; 2 SEALS, Randwick, Australia

## 

Women undergoing risk-reducing salpingo-oophorectomy through the Prince of Wales Hospital Hereditary Cancer Clinic are offered enrolment in a prospective study of bone mineral density (BMD), markers of bone metabolism and cardio-vascular risk factors. Participants are invited to enroll a friend who has ovaries in situ and are aged within five years of the participant as a control population.

Lumbar spine (LS), femoral neck (FN) and total body (TB) bone mineral density changes as a percentage annual loss are reported and compared with age matched norm. Women who were pre-menopausal at the time of oophorectomy have been compared with those who were either already post-menopausal at oophorectomy or had ovaries in situ to evaluate the impact of this intervention.

59 women have been enrolled and have now had 2 or more bone density evaluations – 44 cases and 15 controls. 7 women who were taking HRT or drugs affecting bone metabolism at entry were excluded from this analysis. The average age of women who had undergone pre-menopausal RRSO was 48.4yrs and 49.4yrs for the comparison group.

Of those who were still menstruating at the time of RRSO and were not taking HRT at enrolment, the average annual decrease in LS BMD from year 1 to year 2 was 1.3% of enrolment value, in FN was 0.95% and in TB BMD was 0.6%.The corresponding decrease for the comparison group was 0.88% in LS, 1.3% in FN and an increase of 0.26% for TB. Both groups are aged under 50 and demonstrate a greater than average BMD loss compared with age matched norms (population average annual LS loss is 0.35% in pre-menopausal women and 0.9% for women in early menopause)[1].

The small cohorts so far do not demonstrate any statistically significant differences, but recruitment is ongoing. These findings are consistent with established data which has shown that LS has the greatest loss of BMD in the early menopausal years, with stable or even increasing whole total body BMD^1^.

Data will be presented on total body fat and fat distribution changes in these cohorts.

## References

[B1] Warming Osteoporosis Int 2002 13 105 112 10.1007/s001980200001 11905520

